# Supplementary material for: Collaborative intelligence in AI: Evaluating the performance of a council of AIs on the USMLE
Source: PLOS Digit Health. 2025 Oct 9;4(10):e0000787. doi: 10.1371/journal.pdig.0000787 (PMC12510544; doi:10.1371/journal.pdig.0000787)
Supplement: S2 File — (ZIP) [file pdig.0000787.s002.zip › S2 Data Raw data files containing unprocessed question inputs and LLM outputs including transcripts of deliberations/STEP3_Question-83-1_Type-MC-NJ.docx]

**Facilitator** **[Round 1]:** A 47-year-old man comes to the office to establish care. He recently moved to the area and has not been evaluated by a physician for more than 3 years. He reports a 1-year history of bilateral knee pain that worsens after prolonged standing, but he otherwise has felt well. Medical history is unremarkable and his only medication is acetaminophen as needed for his knee pain. Family history is significant for hypothyroidism in his mother and myocardial infarction in a paternal uncle at age 55 years. The patient drinks five to six beers weekly and does not smoke cigarettes. BMI is 32 kg/m² . Vital signs are temperature 36.1°C (97.0°F), pulse 78/min, respirations 12/min, and blood pressure 138/89 mm Hg. The patient is not in distress. Physical examination discloses no abnormalities. Results of fasting serum lipid studies obtained in preparation for today's visit are shown: Cholesterol Total 264 mg/dL HDL 54 mg/dL LDL 170 mg/dL Triglycerides 200 mg/dL Which of the following is the most appropriate next step in management regarding the patient's laboratory study results? (A) Prescribe atorvastatin (B) Prescribe cholestyramine (C) Recommend diet and low-impact exercise (D) Refer the patient to a cardiologist (E) Repeat fasting laboratory studies in 1 month

**AI_E:** This question pertains to the appropriate clinical management of a 47-year-old man who came in for routine exams. Most importantly, the patient's laboratory results indicate a high total cholesterol (264 mg/dL), High-Density Lipoprotein (HDL) (54 mg/dL), Low-Density Lipoprotein (LDL) (170 mg/dL), and Triglycerides (200 mg/dL). Notably, his LDL and total cholesterol levels are above the preferred range, suggesting dyslipidemia. The normal range for total cholesterol is less than 200 mg/dL, and for LDL, the desirable level is less than 100 mg/dL. His history of knee pain and being overweight (BMI 32 kg/m²), combined with a family history of heart disease and hypothyroidism, adds complexity to his health status. Upon evaluating the options, it's essential to consider his lipid panel, established risk factors for cardiovascular disease, and lifestyle. Statins like atorvastatin or bile acid sequestrants such as cholestyramine indeed help reduce LDL levels, but it might not be the first-line management approach. Recommending a diet and low-impact exercise could be appropriate to address his weight issues and the cholesterol level. Referring the patient to a cardiologist might see premature, given his overall clinical picture. Lastly, repeating fasting laboratory studies in one month could be considered, but given the high levels, waiting may not be the best approach. Drawing from clinical knowledge, diet and physical activity changes are the initial steps for managing dyslipidemia and overweight conditions. These changes not only aid in weight reduction but also help moderate cholesterol levels. With a familial history of heart disease, it becomes even more crucial to implement lifestyle alterations early before resorting to pharmacotherapy. Therefore, the best clinical approach in this scenario would be to advise changes in diet and recommend engaging in low-impact exercises. Such exercises will be better suited for the patient considering his ongoing knee pain complaints. This approach will handle the patient's weight management, knee pain tied to long-standing durations, and dyslipidemia.+

**AI_C:** (1) RESTATING THE QUESTION: The patient, a male of 47 years, who has recently relocated and has not been in contact with a healthcare provider for over three years, presents concerns of persistent bilateral knee pain over the past year, aggravated by excessive periods of standing, but otherwise in a generally good state of health. No significant medical history is noted except for the occasional use of over-the-counter acetaminophen for knee pain relief. His family history reveals occurrences of hypothyroidism (in his mother) and heart attack (in his paternal uncle). He consumes five to six beers per week and refrains from smoking. His BMI indicates obesity, and his pressure readings show elevated numbers. Furthermore, he recently received his serum lipid tests results which reveal high total cholesterol, LDL cholesterol, and triglyceride levels. HDL is within the normal range though. We are to decide the most appropriate next action in managing this patient based on the given lab values. (2) PLANNING: The assumptions to consider include the patient's likelihood of developing atherosclerotic cardiovascular disease (ASCVD), given the family history of early onset myocardial infarction, raised BMI, and the lipid panel results. Alternate assumptions could be the need for lifestyle modifications or pharmacotherapy (statin therapy). Let's evaluate and hypothesize. Since the patient's LDL cholesterol levels are high, we may need to prioritize the assumption about the need for pharmaceutical intervention. (3) CLINICAL REASONING: The lipid panel values indicate that the patient falls into the category of high LDL cholesterol (≥160 mg/dl), and the total cholesterol and triglyceride levels are also elevated. This, combined with his family history of early onset myocardial infarction and his overweight status, are all risk factors for ASCVD. Given these factors, an appropriate clinical thought process would be to implement interventions which can reduce this ASCVD risk. (4) CONCLUDING: After interpreting the lab values in the context of the patient's history and physical, it can be concluded the patient is at high risk for ASCVD and likely requires pharmacological intervention in addition to lifestyle modifications. (5) RESPONSE: Therefore, the most appropriate next step in the management of this patient's laboratory study results would be to prescribe atorvastatin (A). Atorvastatin is a high-intensity statin that will help lower LDL cholesterol levels, possibly reducing the risk of ASCVD. It's important to note that lifestyle modifications should be implemented in addition to this prescription. This comprehensive approach to patient care often yields the best results.

**AI_B:** (1) RESTATING THE QUESTION: The patient in question, a 47-year-old man, has recently moved and has not visited a physician in over three years. Apart from notable pain in his knees after long periods of standing, he reports being healthy except for a family history of hypothyroidism and myocardial infarction. He drinks moderately, doesn't smoke, and has a BMI of 32 kg/m², which is categorized as obese. His vital signs are all within the normal range. The results of his recent fasting serum lipid studies display elevated levels of total cholesterol (normal<200 mg/dL), LDL cholesterol (normal<100 mg/dL), and triglycerides (normal<150mg/dL), but normal HDL levels (normal >40 mg/dL for men). We're tasked with deciding the best course of action to address the abnormal lipid profile results. (2) PLANNING: The assumption here is that the patient’s elevated cholesterol levels, specifically his LDL and triglycerides, need to be addressed given his family history of heart disease and his obesity. One alternate assumption is that lifestyle modification first before medical intervention could positively influence the lipid levels without the side effects of drug intervention. (3) CLINICAL REASONING: Clinically, high LDL cholesterol levels, as seen in this case, contribute to the development and progress of atherosclerosis, which can lead to coronary heart disease. The patient's family history of cardiovascular disease increases his risk of experiencing similar issues, especially considering his relatively high intake of alcohol, overweight status, and lack of physical activity, all known risk factors. However, before considering medical treatment with statins like atorvastatin or cholestyramine, which may present side effects, it's often recommended to first resort to lifestyle changes like diet and exercise which can have positive impacts on these numbers. (4) CONCLUDING: This involves diet modification, like limiting dietary cholesterol intake, and introducing low-impact exercise, which might help his knee pain as well. This step may be the most appropriate considering the patient's overall health and personal history. Given these facts and the lack of other present physical symptoms, referral to a cardiologist or immediate drug prescription may not be necessary as first steps. (5) RESPONSE: So, the most appropriate next step in management regarding the patient's laboratory study results would be (C) Recommend diet and low-impact exercise.

**AI_D:** RESTATING THE QUESTION: This query presents a scenario of a 47-year old man who recently relocated and hasn't seen a physician in over three years. He complains of bilateral knee pain that intensifies after standing for a long time and uses only acetaminophen to manage this pain. Family history reveals hypothyroidism in his mother and a paternal uncle suffering a myocardial infarction at 55 years. The patient consumes five to six beers per week, does not smoke, has a BMI of 32 kg/m² and vital signs within the normal range. He is not in distress, and there are no abnormalities on the physical examination. Fasting blood analysis reveals a total cholesterol of 264 mg/dL, HDL of 54 mg/dL, LDL of 170 mg/dL, and triglycerides of 200 mg/dL. The query seeks the most appropriate management step regarding these laboratory results. PLANNING: The current state of the patient's health revolves around two related issues: elevated lipid levels and overweight (as indicated by his BMI). The cholesterol levels are divided into HDL (good cholesterol), LDL (bad cholesterol), triglycerides, and total cholesterol. Ideal levels are: total cholesterol below 200 mg/dL, LDL below 100 mg/dL, HDL 60 mg/dL or higher, and triglycerides below 150 mg/dL. Therefore, the main issues requiring priority are his overall cholesterol, LDL, and triglycerides levels, which are all higher than preferred values. The management alternatives include prescribing atorvastatin (A) to lower the LDL cholesterol, prescribing cholestyramine (B) which is a bile acid sequestrant that limits absorption of cholesterol from the diet, recommending diet and low-impact exercise (C) as preventative measures, referring the patient to a cardiologist (D) for more specialized care, or repeating fasting laboratory tests after one month (E) to confirm the readings. CLINICAL REASONING: Atorvastatin and cholestyramine could be effective in managing the lipid levels but they should ideally be considered when lifestyle alterations, such as dietary changes and exercise, have failed or are not possible due to other health concerns. At this point, the patient doesn't have symptomatic cardiovascular disease, and prescribing drugs seems premature. The patient's BMI indicates overweight, which can contribute to the increased LDL cholesterol and triglycerides. Therefore, recommending diet and low-impact exercise could address both issues. Referral to a cardiologist is not warranted at this stage as first-line management can be employed by primary care providers. Moreover, repeating the laboratory studies after one month won't resolve the current issue; it would only confirm the existing lipid profile. CONCLUDING: By connecting these reasonings, it can be determined that recommending diet and low-impact exercise (C) would be a substantial primary care preventative measure that can address both the patient's weight and lipid levels. This suggestion does not only help lower cholesterol levels but also potentially reduce the risk for developing cardiovascular disease. It should constitute the first choice since the patient did not have a previous history of high cholesterol or any signs of cardiovascular disease. After following lifestyle modification, the patient's cholesterol levels can be rechecked to ascertain the effectiveness of the intervention. RESPONSE: Therefore, the most appropriate next step in managing this patient regarding his laboratory results is to recommend diet and low-impact exercise (option C). It's recognized as a first-line intervention to reduce cholesterol levels and weight, therefore decreasing the risk of cardiovascular disease. Medication or specialized care is usually considered if lifestyle changes prove ineffective or are not feasible due to other health concerns.

**AI_A:** RESTATING THE QUESTION: The patient in question, a 47-year-old male, has recently relocated and seeks medical care after an absence of over three years. He reports persistent bilateral knee pain of one year's duration, which is aggravated by lengthy periods of standing. His medical records indicate no notable health concerns apart from the use of acetaminophen for knee pain. There are important medical conditions in his family history, namely his mother's hypothyroidism and his paternal uncle's myocardial infarction, which occurred prematurely at age 55. The patient drinks alcohol moderately, abstains from tobacco smoking, and is overweight with a BMI of 32 kg/m². His vital signs are generally normal, except for borderline hypertension. Despite his knee pain, onsite examination, and recent laboratory test results reveal a high cholesterol level. The management options presented for consideration focus on the patient's elevated lipid levels. They include prescribing atorvastatin or cholestyramine, recommending dietary changes and low-impact exercise, referring the patient to a cardiologist, or repeating the fasting laboratory tests in one month. PLANNING: Making any assumption, I will first clarify some key terms. In this case, the LDL, HDL, triglycerides, and total cholesterol levels represent the major components of a standard lipid profile, a test often used to ascertain the risk of coronary heart disease. LDL or "bad cholesterol" has a major role in the atherosclerotic process, thus it's usually viewed negatively when elevated. On the contrary, HDL or "good cholesterol" has protective roles, hence a high level is usually seen positively. Less clear are the roles played by triglycerides and total cholesterol, which usually co-vary with other lipid levels. By asking whether the only abnormality in this patient - an increased lipid profile - warrants pharmaceutical intervention, exercise and diet changes, or additional diagnostic efforts, we can generate alternative assumptions. For instance, the question could assume that medications (atorvastatin or cholestyramine) are needed because the patient's lipid profile is abnormal. However, the guidelines for initiating medication for managing high cholesterol levels are quite specific and not solely based on presence of abnormal lipid levels. Another assumption might be that a diet and exercise (low-impact, in this case, possibly due to the patient's knee pain) are what this patient needs, implemented as a first-line strategy, because this patient is obese. Another could be that further clarification is needed, as perhaps these results may be temporary or imprecise, hence the suggestion of repeating the fasting laboratory tests after a month. Or, because of the family history of cardiovascular disease, perhaps this patient requires a specialist's opinion and should be referred to a cardiologist. CLINICAL REASONING: Having defined the key terms and assumptions, I can then delve into prioritizing them based on clinical significance for the patient's care. The most relevant factors in this case are the patient's family history, BMI, blood pressure, cholesterol levels, and knee pain which restricts his physical activity. Multiple guidelines suggest lifestyle modification is commonly recommended as the initial treatment for patients without overt vascular disease but with risk factors for atherosclerosis, especially when they have modifiable risk factors such as obesity and lower physical activity. So exercise and dietary interventions might be optimal to pursue in this case notwithstanding his knee pain, low impact exercises might be beneficial. On the other hand, the assumption to start with drug therapy is less valid. Even though his cholesterol levels are outside the normal range, they do not sufficiently meet the criteria that warrant initiating statins without the presence of risk factors or established vascular disease whereas the cholesterol absorption inhibitor cholestyramine is generally not a first-line agent in most cases. Referral to a cardiologist seems not an immediate priority as no overt heart disease presents and cardiovascular risks should ideally be managed at the primary care level at this stage. Finally, repeating lab tests is important if there are uncertainties surrounding the initial test results but in this case, there aren't any, hence avoiding unnecessarily repeated tests would be appropriate. CONCLUDING: Thus, for this patient whose most significant problem is high cholesterol levels but who has no manifest vascular disease, intervening with lifestyle modifications would give him a good chance of improving his cholesterol levels as well as positively affecting his other health issues. Exercise especially can help control weight, decrease blood pressure, improve glycemic control, and triglycerides, and elevate HDL cholesterol levels. This is more favorable than initiating medicines right away or seeking specialist referral, as those may be more relevant for individuals with more severe risk factors. RESPONSE: (C) Recommend diet and low-impact exercise

**Facilitator** **[Round 2]:** SUMMARIES: AI_A: The council member suggests that lifestyle modifications, including diet and low-impact exercise, are the best initial approach for the patient. They argue that the patient's cholesterol levels, while elevated, do not meet the criteria for initiating statin therapy, and that the patient's cardiovascular risks should be managed at the primary care level at this stage. AI_B: This council member also recommends lifestyle modifications, including diet and low-impact exercise, as the first step in managing the patient's elevated cholesterol levels. They argue that these changes can have a positive impact on the patient's health without the side effects of drug intervention. AI_C: This council member suggests that the patient's high risk for atherosclerotic cardiovascular disease (ASCVD) warrants pharmacological intervention in addition to lifestyle modifications. They recommend prescribing atorvastatin, a high-intensity statin, to lower the patient's LDL cholesterol levels. AI_D: This council member recommends diet and low-impact exercise as the first-line intervention to reduce the patient's cholesterol levels and weight. They argue that medication or specialized care should only be considered if lifestyle changes prove ineffective or are not feasible due to other health concerns. AI_E: This council member also recommends lifestyle modifications, including diet and low-impact exercise, as the initial steps for managing the patient's dyslipidemia and overweight conditions. They argue that these changes are crucial to implement early before resorting to pharmacotherapy. KEY DIFFERENCES: The key difference in the council members' perspectives lies in the approach to managing the patient's elevated cholesterol levels. AI_A, AI_B, AI_D, and AI_E all recommend lifestyle modifications as the first step, arguing that these changes can have a positive impact on the patient's health without the side effects of drug intervention. On the other hand, AI_C suggests that the patient's high risk for ASCVD warrants pharmacological intervention in addition to lifestyle modifications. QUESTION TO CLARIFY DISAGREEMENTS: Given the patient's high cholesterol levels, family history of heart disease, and obesity, what specific criteria or considerations lead you to recommend lifestyle modifications over pharmacological intervention, or vice versa?. Based on your preceding response, how would you answer the original question: [A 47-year-old man comes to the office to establish care. He recently moved to the area and has not been evaluated by a physician for more than 3 years. He reports a 1-year history of bilateral knee pain that worsens after prolonged standing, but he otherwise has felt well. Medical history is unremarkable and his only medication is acetaminophen as needed for his knee pain. Family history is significant for hypothyroidism in his mother and myocardial infarction in a paternal uncle at age 55 years. The patient drinks five to six beers weekly and does not smoke cigarettes. BMI is 32 kg/m² . Vital signs are temperature 36.1°C (97.0°F), pulse 78/min, respirations 12/min, and blood pressure 138/89 mm Hg. The patient is not in distress. Physical examination discloses no abnormalities. Results of fasting serum lipid studies obtained in preparation for today's visit are shown: Cholesterol Total 264 mg/dL HDL 54 mg/dL LDL 170 mg/dL Triglycerides 200 mg/dL Which of the following is the most appropriate next step in management regarding the patient's laboratory study results? (A) Prescribe atorvastatin (B) Prescribe cholestyramine (C) Recommend diet and low-impact exercise (D) Refer the patient to a cardiologist (E) Repeat fasting laboratory studies in 1 month]

**AI_E:** RESTATING THE QUESTION: This patient, a 47-year-old man with a BMI of 32 kg/m², has been experiencing bilateral knee pain and displays lipid panels with heightened cholesterol levels. The medical management options presented include prescribing medication (atorvastatin or cholestyramine), recommending diet and low-impact exercise, referring the patient to a cardiologist, or repeating fasting laboratory studies in a month. PLANNING: Critical factors for consideration include the patient's age, his obesity (evidenced by his BMI), his elevated cholesterol levels and his familial history of heart disease. These factors could indicate a higher risk for atherosclerotic cardiovascular disease (ASCVD). However, the decision to incorporate pharmacotherapy into his management should be predicated on an evaluation of his overall ASCVD risk, which involves more than just his cholesterol levels. CLINICAL REASONING: The patient’s overall cardiovascular risk should be evaluated using a recognized scorecard like the ACC/AHA ASCVD Risk Score which takes into consideration age, gender, cholesterol levels, blood pressure, and smoking status amongst others. Current cholesterol guidelines recommend initiation of statin therapy if the 10-year ASCVD risk is 7.5% or higher. Considering the mentioned factors the patient's 10-year ASCVD risk might not be greater than 7.5%, hence statin therapy might not be warranted at the moment. CONCLUDING: Given that the patient's cardiovascular risk may not be sufficiently high to warrant pharmacological intervention, the suggested next step would be to recommend lifestyle modifications. These include dietary changes and low-impact exercise, which can help lower cholesterol levels and manage the patient's weight, thus reducing the overall ASCVD risk. RESPONSE: Hence, in response to the question, the most suitable next step in managing this patient's condition would be (C) Recommend diet and low-impact exercise.

**AI_B:** RESPONSE: The question aims to identify the most appropriate next step in managing the patient's laboratory results, which show elevated cholesterol levels. It's important to understand key terms and concepts before further analysis. LDL (low-density lipoprotein), often considered the 'bad' cholesterol, is of concern here, at 170 mg/dL which is considerably high. HDL (high-density lipoprotein) on the other hand, is within the normal range of 54 mg/dL; this is 'good' cholesterol, in the sense that it helps remove LDL from the bloodstream. Also, his BMI (Body Mass Index) of 32 kg/m² indicates that the patient is obese. Consider also his family history of heart disease, and the impact of his lifestyle behaviors such as alcohol consumption and sedentary lifestyle on his health. The main assumption appears to be the presumption that elevated cholesterol levels, in conjunction with other cardiovascular risk factors (like BMI, family history and a somewhat unhealthy lifestyle), might necessitate immediate pharmacologic intervention. An alternative assumption might be that the patient's elevated cholesterol levels and obesity can be managed effectively initially through lifestyle modifications such as a healthier diet and regular low-impact exercise. Questioning each assumption, one should consider: are the cholesterol levels so elevated that immediate medication is necessary? How effective could lifestyle changes be as a primary intervention? How imminent is the threat of atherosclerotic cardiovascular disease (ASCVD) due to these specific parameters, and how may statins contribute or ameliorate such risks? Consider clinical guidelines for initiating statin therapy, which usually include patients with a greater than 7.5% 10-year ASCVD risk. Given the patient's history, it's plausible that they might not fall into this high-risk group, therefore, initiating lifestyle modifications could be a priority. On the note of clinical reasoning, the patient's elevated cholesterol levels alongside a history of obesity and a generally unhealthy lifestyle does put him at an increased risk of atherosclerotic disease. As outlined by the American Heart Association's ASCVD risk calculator, medical professionals should consider age, race, gender, HDL, total cholesterol, systolic blood pressure, along with a risk factor of hypertension or diabetes, among others. Given that our patient is attempting to establish care after several years and possesses known risk factors, calculating his ASCVD risk would be helpful. However, there are strong indicators that his risk may not be elevated enough to necessitate statin therapy. Providing a final response, having expounded all these notions and considered various divergences/assumptions, it appears that recommending lifestyle modifications (option C), such as diet and increased low-impact exercise, is the most appropriate next step in managing this patient's high cholesterol and help reduce other cardiovascular risks. Explicit pharmacotherapy, at least at this stage, may not be strictly necessary. Therefore, I stand with AI_A, AI_B, AI_D, and AI_E.

**AI_D:** 1) RESTATING THE QUESTION: The question here is focused on the best method of managing a patient who presents with elevated cholesterol levels and an increased BMI but no other alarming symptoms or distress indicators. He has a family history of heart disease and hypothyroidism, but his medical history is otherwise unremarkable. His physical examination also revealed no abnormalities. The designated options for management include the prescription of atorvastatin or cholestyramine, recommending lifestyle changes, referral to a cardiologist, or repeating the fasting laboratory studies after a month. It's important to understand the patient's situation, health concerns, and the implications of each management option. 2) PLANNING: The main assumptions to address here are: (1) whether the patient's elevated LDL cholesterol levels and increased BMI are serious enough to warrant immediate pharmacological intervention or if they could be managed with lifestyle changes; and (2) whether referral to a specialist or repeating the laboratory studies would be more beneficial. One potential alternate assumption could be that the patient's elevated cholesterol levels and increased BMI are early indications of metabolic syndrome, which would need a multidisciplinary approach including lifestyle changes and pharmacotherapy. 3) CLINICAL REASONING: Given the complexity of the patient's situation, a careful step-by-step examination of his condition and potential management plans is needed. Despite the patient's elevated cholesterol levels, he has not exhibited any common symptoms of heart disease such as angina or shortness of breath. Furthermore, his drinking habits and BMI clearly indicate potential risks for metabolic and cardiovascular diseases. As per the National Cholesterol Education Program Adult Treatment Panel III guidelines, a high LDL cholesterol level of 170 mg/dL, in conjunction with other risk factors, can be an indication for statin therapy. However, they promote lifestyle modifications as the primary approach in initial management of dyslipidemia before starting pharmacotherapy. 4) CONCLUDING: In light of the patient's current condition and risk factors, it seems reasonable to suggest that lifestyle modifications should be the primary focus initially. This can include dietary changes and low-impact exercises, which could help manage the patient's cholesterol levels and weight simultaneously, potentially alleviating his knee pain as well. The necessity for pharmacological intervention can be reassessed if lifestyle modifications fail to improve the patient's condition or are impracticable due to other health concerns. 5) RESPONSE: Given the patient’s overall risk factors including obesity and an elevated LDL level, useful behavior modifications such as diet and exercise (option C) seem to be the appropriate initial management step. This approach allows us to address multiple risk factors simultaneously while providing the opportunity for the patient to be an active participant in managing his health. Should these interventions fail, pharmacological measures can be considered.

**AI_C:** 1) RESTATING THE QUESTION: The question entails deciding the most appropriate next step in managing the patient's high blood cholesterol levels. The patient, a 47-year-old male, has presented with various risk factors, such as obesity, a history of bilateral knee pain, significant family history including hypothyroidism in his mother and heart disease in a paternal uncle, and a sedentary lifestyle. His latest laboratory study report reveals elevated levels of total cholesterol, low-density lipoprotein (LDL), and triglycerides (TG). 2) PLANNING: The main assumption in this scenario is that the patient's elevated cholesterol levels are a significant risk factor for cardiovascular diseases and should be managed. Alternative assumptions could be that the patient's lifestyle is perhaps more critical in building up this cholesterol_level or that the cholesterol levels might not be problematic if other cardiovascular health metrics are in healthy ranges. Evaluating these assumptions in light of the established lipid management guidelines will help determine the best next step for managing the patient's condition. 3) CLINICAL REASONING: Based on the patient's age, family history of heart disease, and elevated total, LDL cholesterol and TG levels, he is likely at a higher risk of developing cardiovascular disease. These risk factors, along with the patient's obesity (BMI of 32 kg/m²), can contribute to atherosclerosis and subsequent cardiovascular complications without proper management. Given this, the question arises whether to prescribe pharmacotherapy like atorvastatin to help manage the patient's cholesterol levels or to recommend lifestyle modifications. Both interventions aim to reduce LDL cholesterol, the primary target of therapy. 4) CONCLUDING: Based on cardiovascular risk factor analysis, initiation of statin therapy (specifically high-intensity, as indicated by atorvastatin option), is generally considered for LDL-C levels 190 mg/dL or above or a calculated 10-year atherosclerotic cardiovascular disease (ASCVD) risk of 7.5% or higher based on the predominant risk calculator. Evidently, the patient's LDL level exceeds the threshold, which generally is an indication for statin therapy. However, LDL levels can sometimes be artificially inflated by high triglyceride levels, which might not necessitate the statin therapy immediately. 5) RESPONSE: Given that the patient's LDL cholesterol is significantly high, a combination of interventions might be the best course of action. This includes recommending a diet and low-impact exercise (Option C) to help manage obesity and improve overall cardiovascular health. Tropical foods are a good start to lower cholesterol levels and together with low-impact exercise can help improve long-term cardiovascular health. However, considering the patient's high LDL levels, prescribing atorvastatin (Option A) could be crucial in the near term to reduce potential cardiovascular risk. Therefore, I contradict the majority of council members' recommendation and instead agree with AI_C's approach to cholesterol management for this patient.

**AI_A:** RESTATING THE QUESTION: This scenario employs a patient, a 47-year-old man, who has recently established care after a long period without healthcare oversight. He presents with knee pain and is overweight. Lab results indicate that his cholesterol levels are high. His family has a medical history of hypothyroidism and myocardial infarction. We are tasked with determining the most appropriate next step in the patient's management, taking into account his specific conditions and lab results. PLANNING: Many of the assumptions here involve the weight of each factor in the overall health risk assessment for the patient and how to prioritize the treatment approach. We are dealing with a clear case of dyslipidemia, but at the same time, the patient suffers from obesity and knee pain that impacts his functionality. We must question the best approach and whether lifestyle modifications such as exercise and diet alone would suffice at this stage or whether pharmacological interventions (statins in particular) are necessary to mitigate the cardiovascular risks. Another consideration is the need for immediate cardiology consult or repeating the lipid profile after some time. CLINICAL REASONING: The patient is obese with a BMI of 32 kg/𝑚², which is considered in the obese range. Obesity can increase the risk of atherosclerotic cardiovascular disease and can contribute to high cholesterol, as seen in the patient. LDL (low-density lipoprotein) cholesterol levels are significantly high, which is associated with an increased risk of coronary heart diseases. It's important to remember that statins (such as atorvastatin) are indeed the first line of pharmacological intervention for reducing LDL cholesterol levels. However, before jumping to pharmacotherapy, it could be considered to embark on lifestyle modifications first, which are beneficial not only for dyslipidemia but also address the issue of obesity. A prospective plan of diet combined with low-impact exercise (to accommodate his knee pain) may be the most appropriate way to manage the patient's overall health, including his obesity, elevated blood pressure, and dyslipidemia. CONCLUDING: Given these clinical reasonings, while it would be appropriate to prescribe statins such as atorvastatin in some cases of dyslipidemia, it might not be the most appropriate first step for this particular patient. Instead, an approach that aims to modify the lifestyle of the patient (dietary and low-impact exercise) seems to address most of the patient's risk factors. It's also important to consider the patient's values and preferences, especially since adherence to lifestyle changes is heavily dependent on patient motivation. RESPONSE: Therefore, the most appropriate next step in the management of this patient, considering his laboratory study results, should be recommending dietary improvements and an exercise program (option C). This would provide a comprehensive approach, targeting not just the dyslipidemia but also other risk factors like obesity and hypertension. Pharmacological interventions or a specialist referral would perhaps become necessary if these lifestyle modifications aren't successful after a reasonable period or if the patient's condition worsens.

**Facilitator** **[Round 3]:** Summary: AI_A suggests that lifestyle modifications, including diet and low-impact exercise, should be the first step in managing the patient's condition. This approach addresses the patient's obesity, elevated blood pressure, and dyslipidemia. Pharmacological interventions or specialist referrals may be necessary if these modifications are unsuccessful after a reasonable period or if the patient's condition worsens. AI_B agrees with AI_A, recommending lifestyle modifications as the most appropriate next step. AI_B emphasizes that the patient's cholesterol levels and obesity can be managed effectively initially through lifestyle modifications. Pharmacotherapy may not be strictly necessary at this stage. AI_C, however, suggests a combination of interventions. While agreeing with the lifestyle modifications, AI_C also recommends prescribing atorvastatin due to the patient's high LDL levels, which could reduce potential cardiovascular risk. AI_D concurs with AI_A and AI_B, suggesting lifestyle modifications as the primary focus initially. The necessity for pharmacological intervention can be reassessed if lifestyle modifications fail to improve the patient's condition. AI_E aligns with AI_A, AI_B, and AI_D, recommending lifestyle modifications as the most suitable next step. AI_E notes that the patient's cardiovascular risk may not be sufficiently high to warrant pharmacological intervention at this stage. Key Differences: The main difference in perspectives lies in the approach to pharmacological intervention. AI_A, AI_B, AI_D, and AI_E believe that lifestyle modifications should be the first step, with pharmacological intervention considered only if these modifications fail. In contrast, AI_C suggests a combination of lifestyle modifications and immediate pharmacological intervention due to the patient's high LDL levels. Question for Council Members: What specific factors or considerations led you to prioritize lifestyle modifications over immediate pharmacological intervention (or vice versa in the case of AI_C)?. Based on your preceding response, how would you answer the original question: [A 47-year-old man comes to the office to establish care. He recently moved to the area and has not been evaluated by a physician for more than 3 years. He reports a 1-year history of bilateral knee pain that worsens after prolonged standing, but he otherwise has felt well. Medical history is unremarkable and his only medication is acetaminophen as needed for his knee pain. Family history is significant for hypothyroidism in his mother and myocardial infarction in a paternal uncle at age 55 years. The patient drinks five to six beers weekly and does not smoke cigarettes. BMI is 32 kg/m² . Vital signs are temperature 36.1°C (97.0°F), pulse 78/min, respirations 12/min, and blood pressure 138/89 mm Hg. The patient is not in distress. Physical examination discloses no abnormalities. Results of fasting serum lipid studies obtained in preparation for today's visit are shown: Cholesterol Total 264 mg/dL HDL 54 mg/dL LDL 170 mg/dL Triglycerides 200 mg/dL Which of the following is the most appropriate next step in management regarding the patient's laboratory study results? (A) Prescribe atorvastatin (B) Prescribe cholestyramine (C) Recommend diet and low-impact exercise (D) Refer the patient to a cardiologist (E) Repeat fasting laboratory studies in 1 month]

**AI_A:** Restating the question: The case presents a 47-year-old man suffering from bilateral knee pain and elevated lipid levels, particularly LDL cholesterol and triglycerides. His existing health issues include obesity and slightly high blood pressure. The question seeks the best initial management step concerning his laboratory results, offering five possible solutions. Planning: The appropriate management can hinge on several assumptions: (1) the primary problem is dyslipidemia and needs immediate pharmacological intervention, or (2) the primary issue is overall unhealthy lifestyle, and thus, interventions should first target lifestyle modification. The patient's overall health, including obesity (BMI 32 kg/m²) and slightly high blood pressure, along with his cholesterol levels, need to be considered. We also know that his knee pain worsens after standing for long periods, perhaps suggesting a relation between his weight and knee pain. The question is whether the focus should be on lifestyle modification for broad-spectrum benefits or direct pharmacological intervention aimed at the high cholesterol levels. Clinical Reasoning: High cholesterol can contribute to the development of atherosclerosis, leading to potential cardiac problems. However, the patient's case also includes overweight and blood pressure issue that could exacerbate his health risk. Initially targeting comprehensive risk factors like weight and exercise habits may have broader health benefits, including improving knee pain, weight, and cholesterol. Choose pharmacological intervention may address dyslipidemia but will not improve his overall health condition and potential underlying causes of dyslipidemia like unhealthy diet and lack of exercise. Concluding: Considering the broad range of health risks and potential benefits associated with lifestyle modification, I assume that recommending a diet and low-impact exercise is the most suitable primary approach to managing this patient. This approach is more comprehensive and targets the root causes rather than just controlling symptoms. Response: Given the patient's overall health condition and clinical picture, I would recommend diet and low-impact exercise (Option C) as the most appropriate next step in his management. This plan addresses both his elevated cholesterol and other health risks such as obesity and potentially high blood pressure, offering broader health benefits, including potential improvement in his knee pain.

**AI_C:** 1) RESTATING THE QUESTION: We are discussing a 47-year-old man with a history of knee pain, obesity as defined by a BMI of 32 kg/m², borderline hypertension noted with a blood pressure of 138/89 mm Hg, significant family history of myocardial infarction and hypothyroidism, and a lifestyle that includes the consumption of alcohol. His lipid profile shows elevated total cholesterol, LDL cholesterol, and triglycerides with normal HDL cholesterol levels. We need to establish the most appropriate management strategy considering these findings, with options including the prescription of statins (atorvastatin), a bile acid sequestrant (cholestyramine), lifestyle modifications, referral to a cardiologist, or repeating the fasting lipid studies in a month's time. 2) PLANNING: Key assumptions here include the consideration that this patient's elevated LDL cholesterol and triglyceride levels pose an immediate risk warranting pharmacological intervention. However, an alternative assumption is that a healthier lifestyle, including diet and low-impact exercise, could also help address the issues this patient presents, such as obesity, elevated blood pressure, and dyslipidemia. We also need to weigh both management strategies equally. 3) CLINICAL REASONING: It's essential to remember that lifestyle modifications are the bedrock of managing various conditions, including dyslipidemia, hypertension, and obesity. This can lead to crucial improvements even before pharmacological intervention is considered. Additionally, the patient's BMI and lifestyle appear to play a significant role in his overall health. Taking these into account and considering his knee pain, low-impact exercise could be beneficial. Long-term efficacy of management would be significantly enhanced if the patient can adopt healthier lifestyle practices. 4) CONCLUDING: A thoughtful approach would be recommending lifestyle modifications, consisting of a healthier diet and low-impact exercise usefully targeting his obesity, elevated blood pressure, and dyslipidemia. Medications like atorvastatin can be considered if these alterations prove unsuccessful after a reasonable duration or if there is a worsening of patient conditions. 5) RESPONSE: Based on this careful assessment, I propose 'recommend diet and low-impact exercise' (choice C) as the most appropriate next step in managing the patient concerning his laboratory study results. This approach allows us to address multiple health issues he presents with an intervention that carries minimal risk of complications. Later, if this proves ineffective or he develops other risk factors, prescribing atorvastatin might be considered.

**AI_D:** RESTATING THE QUESTION: The query seeks the most appropriate management step given the patient's lab results. The patient has undesirable lipid profile readings, being over the normal ranges for total cholesterol (ideally under 200 mg/dL), LDL cholesterol (ideally below 100 mg/dL), and triglycerides (ideally less than 150 mg/dL). This dyslipidemia, combined with obesity (indicated by a BMI of 32 kg/m²), and a borderline elevated blood pressure, signifies potential risks for cardiovascular disease. The man experiences bilateral knee pain on prolonged standing, his lifestyle includes moderate alcohol, but no smoking and familial medical history carries relevance of hypothyroidism and myocardial infarction. PLANNING: Key assumptions involve the understanding that elevated cholesterol, especially LDL cholesterol, and obesity, along with lifestyle choices, contribute to increased cardiovascular risks. Alternatively, it could be argued that the elevated cholesterol levels might be due to genetic predisposition, given the family history, or lifestyle habits such as diet and alcohol usage. Based on the given choices, the application of lifestyle modifications, beginning a statin medication, the utility of a bile acid sequestrant, fostering cardiological consultation, or repeating the lab studies, warrant clinical priority consideration. CLINICAL REASONING: In this context, it is important to remember that risk of cardiovascular disease is cumulative, accruing over time with exposure to risk factors. The patient of consideration already presents several modifiable risk factors including obesity, dyslipidemia, slightly raised blood pressure, and an unhealthy diet (presumed from BMI and lipid profile). First-line management for high cholesterol and obesity is lifestyle modification, including dietary changes, regular exercise, weight loss, and moderation of alcohol consumption, before the initiation of drugs like statins. Moreover, lifestyle modifications can help improve knee pain by reducing pressure on the joints. As for the future utility of pharmacological intervention, presence of genetic factors (evident from family history) might make lifestyle modifications insufficient, but it is still the initial approach due to lesser side effects and holistic health benefits. CONCLUDING: Weighing the potential for a varied set of implications, lifestyle modification emerges as a method that can address an array of risk factors and improve overall health. Pharmacological management with statin medication might be warranted but is often reserved as a second-line, after lifestyle changes have been trialed or if there are significant underlying cardiovascular risks at play. RESPONSE: Given the patient's obesity, elevated blood pressure, dyslipidemia, and alcohol habits, the most appropriate next step in management regarding the patient's laboratory study results currently would be to "Recommend diet and low-impact exercise" (choice C). These changes can improve his knee pain associated with standing, reduce weight, and potentially positively impact his cholesterol levels. A revisit to the decision of pharmacological management can be made if these prove inadequate.

**AI_B:** RESTATING THE QUESTION: The question requires us to determine the next best step in the management of a relatively healthy 47-year-old man who has a BMI of 32 kg/m², slightly elevated blood pressure, and dyslipidemia that includes high total cholesterol, LDL, and triglycerides, but an acceptable level of HDL. His only complaint is bilateral knee pain resulting from prolonged standing. He is not currently on medication except for acetaminophen for the knee pain. His family history is significant for hypothyroidism and myocardial infarction. PLANNING: The question assumes that the patient's lipid profile and BMI need to be addressed. It's possible, however, that the knee pain could be associated with the weight problem, suggesting that tackling obesity could provide a more holistic approach. Evaluating the different factors, one might prioritize reducing his cardiometabolic risk since it includes multiple parameters such as BMI, blood pressure, and lipid profile. Improving these measures might also indirectly help with his knee pain, assuming it is due to weight-related osteoarthritis. CLINICAL REASONING: A multifactorial problem like this usually needs a multifactorial approach. Starting off with lifestyle alterations such as diet regulation and low impact exercise would target all these issues concurrently, i.e., obesity, knee pain, dyslipidemia, and hypertension. Although his LDL cholesterol is significantly high at 170 mg/dL (normal is <100 mg/dL), and he has family history of myocardial infarction, he does not have other risk factors such as diabetes or personal history of cardiovascular disease. Therefore, the decision to start medication such as atorvastatin would depend on the calculation of his 10-year atherosclerotic cardiovascular disease (ASCVD) risk score. An ASCVD score of 20% or more is a strong factor in favor of initiating statin therapy. CONCLUDING: The strategy should be to first enable the patient to lose weight, control diet, and increase physical activity to see if the lipid profile improves. If his knee pain is due to osteoarthritis, shedding excess weight might also help reduce his symptoms. Therefore, the next step should be recommendation of diet and low-impact exercise to begin with (Option C). If there's no improvement with these interventions, further diagnostic tests, referrals to specialists, or introduction of medications such as atorvastatin (option A) depending on his ASCVD could be explored. RESPONSE: Given the patient's overall clinical picture, while there are multiple parameters that need improving, the initial approach that might help address most of them together would be lifestyle alterations, specifically dietary modifications and low-impact exercises (Option C). This should not only help improve his cardiometabolic profile but could potentially also offer some relief from his knee pain, if it's indeed due to increased weight putting stress on his knees. This approach aligns with standard guidelines for managing dyslipidemia, which usually propose lifestyle modifications as an initial management step.

**AI_E:** Restating the Question: The question asks about the appropriate next steps in managing a patient's health based on his laboratory study findings. This 47-year-old male patient presents with high cholesterol levels, including increased LDL (bad cholesterol), and obesity as indicated by a BMI of 32 kg/m². Considering he has an unremarkable medical history, except for knee pain, hypothyroidism in his mother, and myocardial infarction in an uncle, the question is which among prescribing medication, diet and exercise, referral to a cardiologist, or repeating the fasting laboratory tests would be the most appropriate next step. Planning: The main issue for this patient is the high levels of cholesterol, particularly LDL, which place him at risk of cardiovascular disease. These abnormal values should be considered against his overall health profile and familial history. The assumptions underpinning the different management options include: (A) his cholesterol can be managed with medication alone, (B) his cholesterol levels require a different medication, (C) he can manage his cholesterol levels with diet and exercise alone, (D) his levels are concerning enough to require a specialist's opinion, and (E) his abnormal readings could be a one-time event, requiring repeat tests for confirmation. Considering his overall profile, it's possible that lifestyle changes could improve his health significantly, not only addressing his cholesterol but also his obesity. Clinical Reasoning: The patient’s lipid profile reveals significantly high total cholesterol, LDL, and triglycerides, values that are all too evident risk factors for atherosclerotic cardiovascular disease. However, since he has no other existing co-morbid conditions or previously diagnosed heart disease, and given his relatively younger age, a comprehensive lifestyle modification approach might be effective at this initial stage. Considering other factors such as the patient's BMI, which indicates obesity, his knee pain that is aggravated by prolonged standing, and a significant family history of hypothyroidism in his mother and myocardial infarction in his paternal uncle, these suggest a higher predisposition to developing heart disease. Addressing his weight through diet and low-impact exercise would not only benefit his lipid profile but would improve his overall health, including the knee pain. In fact, exercise has positive effects on increasing HDL, the good cholesterol, and can help lower blood pressure as well. Furthermore, diet modification can play a crucial role in controlling and reducing cholesterol levels and obesity. A prescription of atorvastatin could indeed help lower the cholesterol levels faster, but the question is if it's immediately necessary given that a "less invasive" option is available that can address more than one of his health issues. It's important to exhaust the less aggressive treatments first before jumping to medications. Concluding: After considering the patient's current health state, though his cholesterol levels are high, they don't seem to warrant immediate pharmacological intervention. His overall health could benefit more from a holistic approach involving lifestyle modifications like diet and low-impact exercises, which would also positively impact his obesity and potentially his knee discomfort. With his BMI and cholesterol in mind, a lifestyle modification focused on diet and exercise should be the most appropriate next step. Response: Considering the information, option (C) Recommendation of diet and low-impact exercise would be the most appropriate next step in the management of this patient's laboratory study results. This approach addresses a broad spectrum of his health issues. It provides initial management for his high cholesterol levels while simultaneously addressing his BMI and potentially his knee discomfort. If these modifications do not provide an adequate response over a reasonable time, a review of the medical management including considering medications like atorvastatin or specialist referrals might be a secondary step to discuss.

**Facilitator** The consensus among the AI responses is that the most appropriate next step in managing the patient's laboratory study results is to recommend diet and low-impact exercise (Option C). This approach is favored due to the patient's overall health profile, which includes high cholesterol levels, obesity (indicated by a BMI of 32 kg/m²), slightly elevated blood pressure, and knee pain that worsens with prolonged standing. The reasoning behind this consensus is that lifestyle modifications, including diet and low-impact exercise, can address multiple health issues concurrently. These include obesity, knee pain, dyslipidemia, and hypertension. While the patient's LDL cholesterol is significantly high, and he has a family history of myocardial infarction, he does not have other risk factors such as diabetes or personal history of cardiovascular disease. Therefore, the decision to start medication such as atorvastatin would depend on the calculation of his 10-year atherosclerotic cardiovascular disease (ASCVD) risk score. The consensus also acknowledges that if lifestyle modifications do not provide an adequate response over a reasonable time, a review of the medical management including considering medications like atorvastatin or specialist referrals might be a secondary step to discuss. However, given the patient's current health state, the consensus is that his cholesterol levels do not warrant immediate pharmacological intervention. Instead, a holistic approach involving lifestyle modifications is recommended as the most appropriate next step.
